# Supplementary material for: Improving Peach Fruit Quality Traits Using Deficit Irrigation Strategies in Southern Tunisia Arid Area
Source: Plants (Basel). 2022 Jun 23;11(13):1656. doi: 10.3390/plants11131656 (PMC9269496; doi:10.3390/plants11131656)
Supplement: Supplementary file 1 [file plants-11-01656-s001.zip › plants-1730125-supplementary.pdf]

Table S1. Single crop coefficients Kc for peach tree.

$K_{c\ ini}$	$K_{c\ mid}$	$K_{c\ end}$
0.55	0.90	0.65

Table S2. Effects of deficit irrigation DI and PRD_{50} on the skin color of peach fruit under different irrigation treatments (FI, DI and PRD_{50}). Different letters refer to significant differences tested by Duncan's multiple range test (P<0.05).

Irrigation Treatments	L^*	a^*	b^*	CI
FI	52.45±1.11a	23.01±7.01a	33.17± 2.35a	13.22± 4.30a
DI	52.47±1.77a	23.41±4.41a	30.71±10.45a	14.52±18.63a
PRD50	51.62±4.39a	22.64±6.87a	33.64±5.72a	13.05± 5.37a

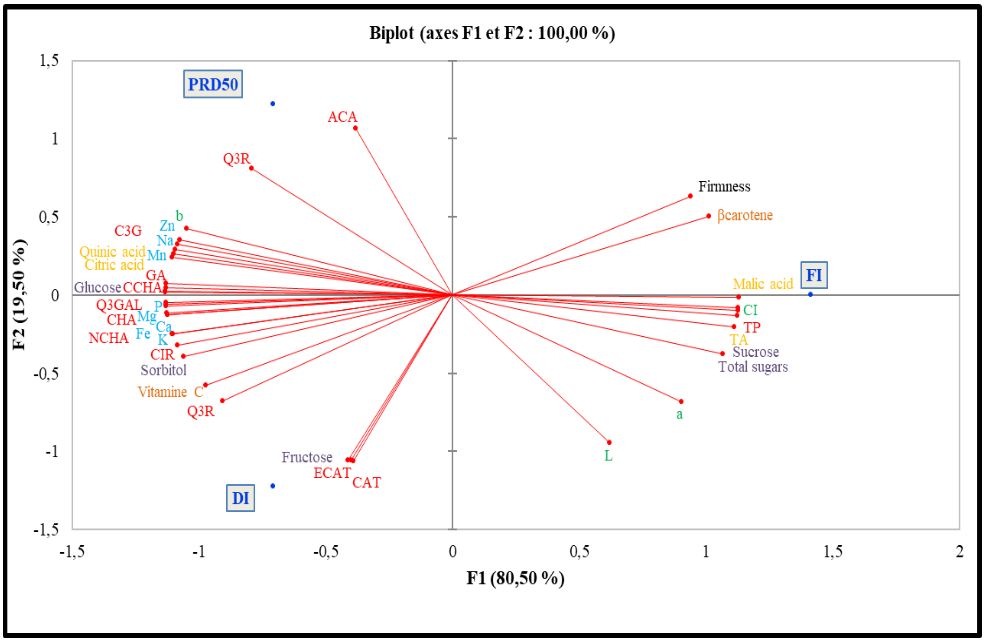

Figure S1. Principal component analysis for the quality traits evaluated on the Flordastar peach irrigated with three irrigation strategies (FI, DI and PRD_{50}). Analysis was performed using mean data of the 2016 growing season.
